# Exploitation of HPLC Analytical Method for Simultaneous Determination of Six Principal Unsaturated Fatty Acids in *Oviductus Ranae* Based on Quantitative Analysis of Multi-Components by Single-Marker (QAMS)

**DOI:** 10.3390/molecules26020479

**Published:** 2021-01-18

**Authors:** Shihan Wang, Yuanshuai Gan, Hong Kan, Xinxin Mao, Yongsheng Wang

**Affiliations:** 1 College of Chinese Medicine Materials, Jilin Agricultural University, Changchun 130118, Jilin, China; kanhong@vip.163.com (H.K.); maomaoheniunai@126.com (X.M.); 2 School of Pharmaceutical Sciences, Jilin University, Changchun 130021, Jilin, China; ganys18@mails.jlu.edu.cn

**Keywords:** *Oviductus Ranae*, UPLC-MS/MS, HPLC, QAMS, unsaturated fatty acids, Taguchi design

## Abstract

As one of the featured products in northeast China, *Oviductus Ranae* has been widely used as a nutritious food, which contains a variety of bioactive unsaturated fatty acids (UFAs). It is necessary to establish a scientific and reliable determination method of UFA contents in *Oviductus Ranae*. In this work, six principal UFAs in *Oviductus Ranae*, namely eicosapentaenoic acid (EPA), linolenic acid (ALA), docosahexaenoic acid (DHA), arachidonic acid (ARA), linoleic acid (LA) and oleic acid (OA), were identified using UPLC-MS/MS. The UFAs identified in *Oviductus Ranae* were further separated based on the optimized RP-HPLC conditions. Quantitative analysis of multi-components by single-marker (QAMS) method was implemented in content determination of EPA, ALA, DHA, ARA and OA, where LA was used as the internal standard. The experiments based on Taguchi design verified the robustness of the QAMS method on different HPLC instruments and chromatographic columns. The QAMS and external standard method (ESM) were used to calculate the UFA content of 15 batches of *Oviductus Ranae* samples from different regions. The relative error (r < 0.73%) and cosine coefficient showed that the two methods obtained similar contents, and the method validations met the requirements. The results showed that QAMS can comprehensively and effectively control the quality of UFAs in *Oviductus Ranae* which provides new ideas and solutions for studying the active components in *Oviductus Ranae*.

## 1. Introduction

*Oviductus Ranae*, derived from dried tubal product of *Rana temporaria chensinensis* David, has the effects of anti-fatigue, nourishing the lungs and improving immunobiological activity [1,2,3]. As a precious natural product in northeast China, *Oviductus Ranae* has been used for nearly thousands of years and has developed into a series of nutrition, health food and traditional Chinese medicine. *Oviductus Ranae* is rich in nutrients, including a variety of unsaturated fatty acids (UFAs), protein, vitamins and steroids [3,4,5,6,7,8,9]. Among them, UFAs, as important bioactive substances, have excellent biological activities in regulating immunity and preventing a series of cardiovascular and cerebrovascular diseases such as hyperlipidemia, hypertension and thrombosis [10,11,12,13,14]. The content of UFA is considered to be closely related to the quality of *Oviductus Ranae* [15]. However, due to the influence of breeding environment, production technology and preservation conditions [2], the content of UFA in *Oviductus Ranae* in different regions varies, resulting in the uneven quality of *Oviductus Ranae* in the market [15]. Therefore, it is necessary to develop an economical and convenient method to monitor the content of UFA in *Oviductus Ranae* to ensure its quality.

Currently, the main method to determine the UFAs in *Oviductus Ranae* is external standard method (ESM) [15]. However, this method requires various corresponding standards and might be limited by uncontrollable factors [16]. In addition, the cost of standards for UFAs with a purity of 98% or above is high [17,18], and UFAs are easy to be oxidized which make it difficult to preserve the standard substances [19,20,21]. Compared with the traditional ESM, the HPLC-DAD chromatographic analysis method based on quantitative analysis of multi-components by single-marker (QAMS) strategy has the potential to reduce those shortcomings. HPLC-DAD and other chromatographic separation and analysis methods combined with QAMS can be used to complete the determination of multiple components in samples using only one standard substance [22,23,24]. This method is suitable for detecting UFAs in the *Oviductus Ranae*. This method is simple, can greatly reduce the detection cost, and has broad application and promotion potential. The QAMS method can calculate the content of other components to be measured by measuring the content of a representative component in a series of similar compounds according to the functional relationship and proportional relationship among various active components in *Oviductus Ranae* [25,26]. This method usually selects the compounds that are readily available, inexpensive, and have significant bioactivity as internal reference materials, and can simultaneously determine other multiple components [27,28].

The aim of these efforts is to develop a new method for simultaneous quantitative determination of the six major UFAs in *Oviductus Ranae* based on the QAMS strategy, which can significantly reduce the detection cost and provide a reference for the regulatory authorities to rapidly monitor the quality of *Oviductus Ranae*. To achieve this goal, this work optimized the detection wavelength, mobile phase and column temperature conditions, established HPLC-DAD separation analysis method for UFAs and identified major UFAs in *Oviductus Ranae* through UPLC-MS/MS. Finally, six major UFAs in *Oviductus Ranae* from different regions were quantitatively determined based on the QAMS strategy, which was used to verify the robustness of the QAMS method on different instruments and chromatographic columns.

## 2. Results and Discussion

### 2.1. Optimization of HPLC Separation Conditions

**Figure 1:**
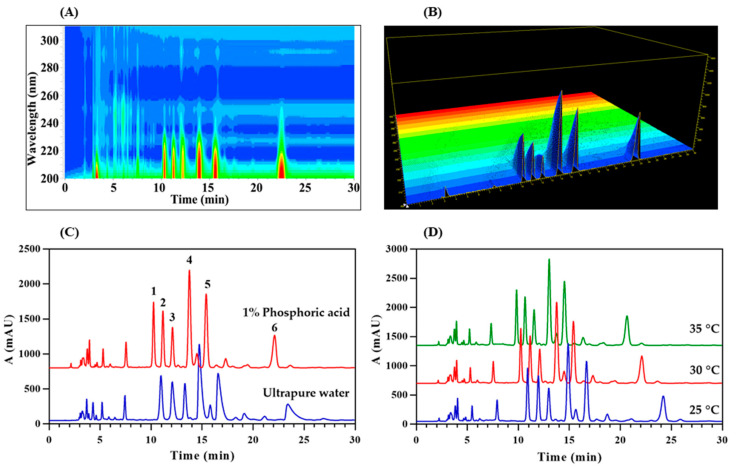
Optimization of HPLC separation conditions: (**A**) Two-dimensional isoabsorption spectra of diode array detector (DAD); (**B**) Three-dimensional absorption spectra of DAD; (**C**) Influence of mobile phase B components on chromatographic peaks; (**D**) The influence of column temperature (25, 30 and 35 °C) on the separation effect of HPLC.

To optimize the analysis conditions of HPLC, the DAD detector was set to scan the spectral information between 200–300 nm (scanning interval: 1 nm) [29]. In the two-dimensional isoabsorption diagram of DAD (Figure 1A), the red area that is approximately elliptical represents the compound with high absorbance. Its center is located at the wavelength of 203 nm, which is suitable for the detection wavelength of the compound. In the three-dimensional absorption map (Figure 1B), it also shows the same result as the two-dimensional isoabsorption map. The peak absorbance of the tested compound appears at 203 nm. The detection wavelength of 203 nm belongs to the end absorption, which is already close to the detection limit of the short wavelength of 190 nm of the DAD detector. This may be due to the relatively simple chemical structure of long-chain UFAs, which only rely on the π-electron transition of carbon-carbon double bonds and carbonyl groups to have weak UV absorption [30]. Severe chromatographic peak tailing occurred with ultrapure water as mobile phase B (Figure 1C). The addition of 1% phosphoric acid as an ion inhibitor can significantly reduce the tailing phenomenon of chromatographic peaks and improve the separation efficiency of the components [31]. Changes in temperature usually change the resolution and peak time of the compound. At three different temperatures (25, 30 and 35 °C) for this work (Figure 1D), as the temperature increases, the peak time of the chromatographic peak gradually decreases. Since higher temperature is not friendly to the chromatographic column, it may damage the stationary phase of the chromatographic column and affect the service life of the chromatographic column. Therefore, on the premise of achieving the separation effect, a moderate 30 °C was selected as the column temperature for analysis. Using optimized HPLC conditions, the resolutions of adjacent chromatographic peaks are all greater than 1.5, and the number of theoretical plates is greater than 8000 based on each chromatographic peak, and the components to be analyzed have reached the ideal separation effect. Compared with the previous HPLC method for the analysis of *Oviductus Ranae* samples [1,5], the newly established method always used a high proportion of organic phase (86–100% nitrile) as the HPLC mobile phase, avoiding the use of 100% water which is more friendly to the chromatographic column. In addition, the HPLC method could quickly complete the HPLC analysis of samples within 30 min, which significantly improved the analysis efficiency.

### 2.2. HPLC Method Validation

The relative standard deviation of the peak area of the chromatographic peak is used to represent the verification result of the methodology, where the smaller the RSD, the more reliable the method is. In six repeated determinations of the same sample, the RSD value of the repeatability of each chromatographic peak was less than 0.35% (Table 1). The sample was placed at room temperature for 24 h, and the peak area RSD value measured at different time points was lower than 0.64%, indicating that the tested sample has good stability. The RSD value of each chromatographic peak area of six independent samples of the same batch prepared separately after continuous injection was less than 0.19%, which proved the good repeatability of the quantitative method. The accuracy of the proposed method was evaluated by adding accurate amounts of each standard to the sample. For the six major UFAs analyzed in *Oviductus Ranae*, their recovery rates are between 99.00–104.14%, and the RSD value is also between 1.33–1.88%, which confirms the good accuracy of the analytical method. All the above method validation data show the reliability of the HPLC method.

### 2.3. UPLC-MS/MS Component Analysis

**Figure 2:**
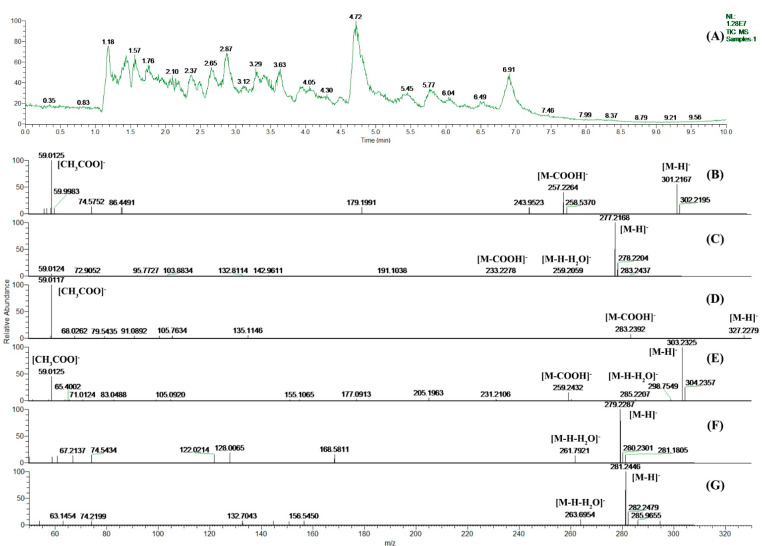
The HPLC and UPLC-MS/MS analysis of principal unsaturated fatty acids (UFAs) in *Oviductus Ranae*: (**A**) The HPLC chromatogram of principal UFAs in *Oviductus Ranae*; (**B**–**G**) are the corresponding MS^2^ mass spectrum of EPA, ALA, DHA, ARA, LA, and OA, respectively.

The total ion chromatogram (TIC) of *Oviductus Ranae* generated by UPLC-MS/MS analysis is shown in Figure 2A. The extracted ion chromatograms (EICs) of *Oviductus Ranae* sample and UFA standards generated are shown in Figures S2 and S3. Combined with the results of the determination of UFAs in the HPLC analysis (Figure 1C), there were 6 (peak 1–6) important chromatographic peaks in the chromatogram, so this work focused on analyzing these 6 chromatographic peaks corresponding to the most important 6 kinds of UFA components. The retention time of the 6 most important chromatographic peaks in HPLC and their corresponding MS^2^ mass spectra inference information are listed in Table 2.

In Figure 2B–G, the molecular ion peak of [M − H]^−^ can be observed, so the relative molecular mass of the corresponding compound can be inferred. In Figure 2B–E, the alpha cleavage of the carboxyl end of the UFA molecule generates a fragment ion peak with *m/z* of [M − COOH]^−^. In addition, it can also be observed that the ion peak with *m/z* of [CH_3_COO]^−^ is the classic oxygen-containing fragment ion peak produced by the cleavage of fatty acid. In Figure 2E–G, the molecular ion ([M − H]^−^) further loses a water molecule, resulting in a classical fragment ion peak with a smaller *m/z* ([M − H − H_2_O]^−^). Finally, compared with the standard spectrums in the NIST Mass Spectral Database and references [3,9], it is speculated that the compounds corresponding to Figure 2B–G are EPA, ALA, DHA, ARA, LA, and OA, respectively.

### 2.4. Relative Correction Factor (RCF)

In this work, LA is a relatively high content of UFA in *Oviductus Ranae*, and it is easy to separate under conventional chromatographic conditions. In addition, LA has a relatively low acquisition cost and excellent health effects in cardiovascular diseases. Therefore, LA (the total LA), which is relatively cost-effective, was selected as an internal reference. In the linear range, the content of UFA is proportional to the peak area. Different injection volumes will cause simultaneous changes in fatty acid content and detector peak area, but theoretically, the relative correction factor is a constant value. Taking the mean value of the equivalent correction factor under different injection volumes as the final result, the RSD value reflects the stability of the calculated result. In order to ensure the accuracy of the relative correction factor as much as possible, four decimal places are reserved uniformly. According to different injection volumes, the mean values of the correction factors of EFA, ALA, DHA, ARA and OA were calculated to be 2.5382, 1.4356, 3.2724, 2.4019 and 0.1998 (Table 3), respectively. Their maximum RSD value is 1.99%, which is less than 2.00%, indicating that the calculated relative correction factor has good stability.

### 2.5. Robustness Test Based on Taguchi Design

**Figure 3:**
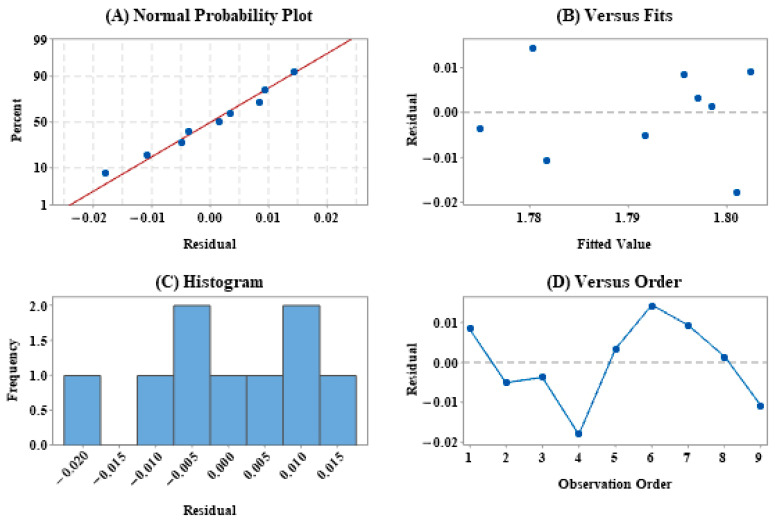
The residual distribution of the mean in the Taguchi design experiment results: (**A**) Normal probability plot; (**B**) Versus fits; (**C**) Histogram; (**D**) Versus order.

Taguchi design is a scientific and effective method for robust design of multi-factor experiments. In this work, in order to evaluate the influence of different HPLC instruments and columns on the robustness of RCF in the QAMS method, Taguchi design–static design (Table 4) was implemented. The analysis was based on the experimental results designed by Taguchi, and the residual distribution of the mean is shown in Figure 3. The normal probability plot of the residual results in Figure 3A shows a good linear distribution, which means that the experimental results designed by Taguchi are normally distributed, and in the histogram (Figure 3C), the residuals are also approximate. Normal distribution means that the experimental data collection is reasonable and sufficient, and the experimental process design is stable. It can be seen from Figure 3B,D that all experimental results are randomly distributed, which indicates that the experimental data has no outliers, which further confirms the robustness of the RCF in the QAMS method.

The results of intuitive analysis in Table 4 show that the average values of the RCF of the five UFAs to be tested on different HPLC instruments and columns are 2.5222, 1.4112, 3.2132, 2.4036 and 0.1988, and their corresponding RSD values are 1.08%, 1.03%, 1.28%, 1.27% and 1.58%. The average value of all RSD values is less than 5%, which indicates that the errors caused by different HPLC instruments and chromatographic columns are very small, which provides a basis for further checking the stability of RCF. The response results shown in Table S1 of the Supplementary Materials indicate that the HPLC instrument is the most important factor affecting the signal-to-noise ratio (Delta = 0.059), followed by the influence of the column (Delta = 0.056), and the column is the most important factor for the mean value (Delta = 0.021). In Table S2 of the Supplementary Material, the results of the analysis of variance of the Taguchi design show that the HPLC instrument and the chromatographic column have insignificant effects on the average value and the signal-to-noise ratio (*p* > 0.05), which confirms the robustness of the RCF in the QAMS method and provides a favorable basis. In this work, the most typical analytical column (250 × 4.6 mm) was used to investigate the effect of different aperture, carbon loading and surface area on the stability of QAMS method. The experimental results of Taguchi design verified the robustness of the method in the application of conventional analytical column (250 × 4.6 mm) to a certain extent.

According to the above analysis results, although different HPLC instruments and chromatographic columns will inevitably affect the RCF of UFAs to be tested in *Oviductus Ranae*, these changes have no significant impact on the results, and the fluctuation range is acceptable. The QAMS method established in this work is robust and laid the foundation for its popularization and application in different laboratories.

### 2.6. Consistency Assessment of QAMS and ESM Results

In order to evaluate the consistency of the results of the QAMS method and the ESM, the two methods were used to determine the content of six major UFAs in 15 batches of *Oviductus Ranae* (Table 5). After 15 batches of samples were verified, the relative errors of these two methods were controlled within 0.73%, and their results showed good agreement. The relative error in the Supplementary Material Table S3 shows that the two methods have the largest difference (0.73%) in determining the content of OA. This may be due to the relatively high content of OA in the *Oviductus Ranae* samples. In most batches of samples, the content of OA is an order of magnitude higher than that of LA as an internal reference. The cosine of the included angle is theoretically between 0 and 1. The closer the value is to 1, the higher the similarity of the results [32,33]. The cosine coefficient of these two methods both show a value close to 1, indicating that there is no significant difference between the calculation results of the ESM and the QAMS method. Therefore, the QAMS method established in this work can very quickly monitor the content of the main UFAs in *Oviductus Ranae*.

## 3. Materials and Methods

### 3.1. Reagents and Samples

Chromatography grade methanol and chromatography grade acetonitrile were purchased from Fisher Scientific (Fair Lawn, NJ, USA). Ultrapure water was obtained from a gradient water purification system (Water Purifier, Sichuan, China). The standard products of eicosapentaenoic acid (EPA), docosahexaenoic acid (DHA) and arachidonic acid (ARA) were purchased from TanMo Quality Testing Technology Co., Ltd. (Beijing, China). The standards of α-linolenic acid (ALA), linoleic acid (LA) and oleic acid (OA) were purchased from ANPEL Laboratory Technologies (Shanghai) Inc. (Shanghai, China). The six standards were verified by HPLC using the peak area normalization method and the purity of the products is greater than 98%. All other chemical reagents were purchased from Beijing Chemical Factory (Beijing, China) and are of analytical grade. *Oviductus Ranae* samples were stored in the Department of Natural Medicine Chemistry, School of Pharmaceutical Sciences, Jilin University. Its sources cover the main producing areas of Changbai Mountain, Jilin Province. The detailed collection information is listed in Table 6.

### 3.2. Preparation of Standard Solutions

The standards of UFAs were dissolved in methanol to prepare mixed standard solutions containing different concentrations. Then the mixture standard solution was diluted to different concentrations (diluted 2–10 times). The diluted solution was passed through a 0.22 μm microporous membrane. The filtrate was used for HPLC analysis and the standard curve was plotted. In the ESM, the standard curve was used to determine the content of UFAs in *Oviductus Ranae*. (Supplementary Materials, Figure S1).

### 3.3. Preparation of Samples

After *Oviductus Ranae* samples were pulverized with a pulverizer (the particle size is controlled at about 1 mm), 0.8000 g *Oviductus Ranae* powder was measured. The samples were then wrapped with Whatman filter paper and placed in a Soxhlet extractor. 70 mL of petroleum ether was added to the round bottom flask and heated to reflux for 6 h. After extraction, the solvent was removed using a rotary evaporator to evaporate under reduced pressure at 45 °C. Finally, the extracts were dissolved in methanol and diluted to 2 mL in a volumetric flask. The solution was passed through a 0.22 μm microporous membrane before HPLC analysis. During the extraction and HPLC detection, degradation of UFAs was not observed.

### 3.4. HPLC Analysis

#### 3.4.1. Development of HPLC Analysis Method

The Agilent 1260 series high-performance liquid chromatograph (Palo Alto, CA, USA) equipped with a quaternary pump, autosampler, warm column chamber and a diode array detector (DAD) was used to perform HPLC analysis of the *Oviductus Ranae* sample. The column model used was Agilent TC-C18 (250 × 4.6 mm, 5 µm), and the injection volume was 10 µL. The flow rate was set to a linear gradient of 1.0–0.5 mL/min for 0–14 min and maintained at 0.5 mL/min for 14–30 min. The gradient elution conditions of the mobile phase were 0–10 min linear gradient 86–93% A, 10–20 min maintain 93% A, 20–30 min linear gradient 93–100% A. The mobile phase A was acetonitrile, and the composition of mobile phase B (water or 1% phosphoric acid aqueous solution) was optimized according to the separation effect of the chromatographic peak. The detection wavelength of the sample was confirmed according to the DAD full-wavelength scan result and the column temperature was optimized to achieve the best separation of the chromatographic peaks. Agilent Chemstation software (Palo Alto, CA, USA) was used to record and process the acquired HPLC data.

#### 3.4.2. Validation of HPLC Analysis Method

The rationality of the HPLC analysis method was verified from the aspects of precision, stability, repeatability and accuracy. Same sample solution was tested at six consecutive times in the same day to verify the precision. The stability of the same sample solution was tested 0 h, 2 h, 4 h, 6 h, 8 h, 10 h, 12 h, and 24 h after preparation. To evaluate the repeatability, six independent sample solutions from the same batch were tested in parallel. The reference substance was added to the sample solution with ratio 1:1 and the sample recovery experiment was performed to determine the accuracy.

### 3.5. UPLC-MS/MS Component Identification

UPLC-MS/MS analysis was performed on a Q-Exactive MS mass spectrometer (Waltham, MA, USA) equipped with a UPLC system and a heated electrospray ionization source (HESI). The column used was ACQUITY UPLC BEH C18 (100 × 2.1 mm, 1.7 μm), and the column temperature was set to 30 °C with mobile phase eluent A (ultrapure water) and eluent B (acetonitrile). The flow rate was 0.4 mL/min and the gradient elution conditions were 0–2 min, linear gradient 86–93% B, 2–4 min keep 93% A, 4–6 min linear gradient 93–100% B, 6–10 min keep 100% B. The negative ion mass spectrometry scan mode was used for detection with a spray voltage at 3.1 kV. The flow rates of the sheath gas, auxiliary gas and sweep gas were set at 10 arbitrary units (arb unit), 15 arb unit, and 0 arb unit, respectively. The capillary temperature and the auxiliary gas heater temperature were 320 °C and 26 °C, respectively. The scan range of *m/z* was 100–1200 Da. The Xcalibur 4.1 workstation was used to collect and record the mass spectrum data.

### 3.6. Development of QAMS Analysis Method

#### 3.6.1. Validation of HPLC Analysis Method

When establishing the QAMS method, the most critical step is to obtain the relative correction factor (RCF) between the internal reference and the peak component to be tested, based on the mixed standard solution, and use it as a constant for the corresponding component to be tested in the sample determination of content [34,35]. In order to obtain the relative correction factor of the compound to be analyzed, a suitable compound needs to be selected as the internal reference. The internal reference should be abundant in the sample, have relatively stable properties, have good biological activity, and its monomer standards should be easy to obtain [28].

According to Equation (1), the relative correction factor of the internal reference LA to be tested component i was calculated.

(1) $$F_{\mathrm{LA}/\mathrm{si}}\;=\;\frac{F_{\mathrm{LA}}}{F_{\mathrm{si}}}\;=\;\frac{A_{\mathrm{LA}}/C_{\mathrm{LA}}}{A_{\mathrm{si}}/C_{\mathrm{si}}}$$

In the equation, F_LA/si_ is the calibration factor of the internal reference LA standard to be tested for the UFA standard, A_LA_ and C_LA_ (mg/mL) are respectively based on the internal reference LA standard in the mixed standard solution peak area and concentration, A_si_ and C_si_ (mg/mL) are respectively based on the peak area and concentration of the UFA standard to be tested in the mixed standard solution.

#### 3.6.2. Determination of UFAs in *Oviductus Ranae* Based on QAMS

Firstly, the content of LA in different batches of *Oviductus Ranae* was determined according to the standard curve of LA, and then QAMS was used to determine the content of UFAs in 20 batches of *Oviductus Ranae*.

According to Equation (2), the concentration of the UFA component in the *Oviductus Ranae* sample was calculated.

(2) $$C_{i}{\;=\;F}_{\mathrm{LA}/\mathrm{si}}{\;\times \;C}_{s}\;\times \;\frac{A_{i}}{A_{s}}$$

In the equation, A_s_ and C_s_ (mg/mL) are the peak area and concentration of LA in the *Oviductus Ranae* sample calculated based on the standard, and Ai and Ci (mg/mL) are the peak area and concentration of UFAs to be tested in the *Oviductus Ranae* sample.

Finally, the content of various UFAs in the *Oviductus Ranae* sample was calculated according to Equation (3).

(3) $${\mathrm{UFA}}_{i}\;(\mathrm{mg}/g)\;=\;\frac{C_{i}\;\times \;V}{m}$$

In the equation, UFA_i_ (mg/g) is the content of UFA in the *Oviductus Ranae* sample, and m (g) is the weight of the *Oviductus Ranae* weighed when the sample solution is made. In this work, it is 0.8000 g, and V is the constant volume of the *Oviductus Ranae* sample solution, which is 2 mL in this work.

### 3.7. Robustness Test Based on Taguchi Design

To assess the established QAMS analysis method in practical application, it is necessary to investigate the robustness of the method. In different laboratories, researchers usually inevitably use different types of HPLC instruments, columns and detectors, which may affect the peak area calculation. This is mainly related to the detector’s slit, bandwidth value, slope sensitivity and peak width, and integration method [22]. Different types of chromatographic columns may have different carbon loading, surface area and end caps, which will affect the symmetry coefficient of the chromatographic peak and the number of theoretical plates.

In this work, Taguchi design, known as “robust design”, was introduced to investigate the robustness of QAMS analysis methods in different types of HPLC instruments and chromatographic columns [22]. Specifically, a two-factor three-level Taguchi design–static design was created in Minitab (Version 19, Minitab, PA, USA). The HPLC instrument (Table S4) and the column (Table S5) were used as two independent control factors (Table 7). The noise factor is the change of the measurement result with the fluctuation of the environment. The response value is the measured value of the calibration factor of the UFA standard to be tested. It has the characteristics of expectation. It is expected that the measured value is close to the true value. The signal-to-noise ratio (S/N) was introduced as an evaluation parameter, with the response value as the target and the effects of mean and variance are considered at the same time. Finally, Taguchi analysis in Minitab was used to explain the experimental results.

### 3.8. Consistency Assessment of QAMS and ESM Results

The QAMS method was applied to determine the content of UFAs in 15 batches of different *Oviductus Ranae* in the main producing area of Changbai Mountain. The measured content of the conventional ESM and the content calculated by QAMS usually adopt the cosine coefficient to evaluate the consistency of the results (Equation (4)) [17,18]. The closer the cosine value of the included angle is to 1, the closer are the measurement results of the two methods [33].

(4) $$S_{i}\;=\;\frac{\sum_{i\;=\;1}^{n}x_{i}y_{i}}{\sqrt{\sum_{i\;=\;1}^{n}{{(x}_{i})}^{2}}\;\sqrt{\sum_{i\;=\;1}^{n}{{(y}_{i})}^{2}}}$$

In the equation, S_i_ is the consistency of the results calculated by the ESM and the QAMS method. x_i_ and y_i_ are the content of UFAs in *Oviductus Ranae* calculated by ESM and QAMS, respectively.

## 4. Conclusions

This work successfully established the separation and analysis method of UFAs in *Oviductus Ranae* by HPLC-DAD and identified six major UFAs through UPLC-MS/MS, including EPA, ALA, DHA, ARA, LA and OA. Finally, based on the QAMS strategy, a new method for simultaneous quantitative determination of six major UFAs in *Oviductus Ranae* was developed and the robustness of the method on different instruments and chromatographic columns was verified by Taguchi design. In the traditional external standards approach, content determination usually requires a variety of standards. The new method established in this work can simultaneously determine the content of six major UFAs in *Oviductus Ranae* by relative correction factor (RCF) using only the relatively cost-effective standard LA as an internal reference. The analysis is quick and simple, which greatly reduces the measurement cost, makes up for the deficiency of the quality control method of *Oviductus Ranae*, and provides a reference for the supervision department to monitor the quality of *Oviductus Ranae* quickly. In addition, the determination technique developed in this work is not limited to *Oviductus Ranae* samples but can be extended to provide theoretical basis for the monitoring of similar components in other natural products.

## Figures and Tables

**Table 1:** The methodological verification of HPLC analytical methods.

Composition	Precision RSD (%)	Stability RSD (%)	Repeatability RSD (%)	Accuracy	
				Recovery Rate (%)	RSD (%)
EPA	0.32	0.64	0.15	101.62	1.60
ALA	0.32	0.41	0.18	99.00	1.51
DHA	0.35	0.44	0.18	101.18	1.62
ARA	0.33	0.20	0.19	100.20	1.49
LA	0.21	0.20	0.16	100.30	1.33
OA	0.27	0.22	0.12	104.14	1.88

EPA: eicosapentaenoic acid; ALA: α-linolenic acid; DHA: docosahexaenoic acid; ARA: arachidonic acid; LA: linoleic acid; OA: oleic acid; RSD: relative standard deviation.

**Table 2:** The retention times of the six most important chromatographic peaks in HPLC and their corresponding MS^2^ mass spectra inference information.

Peak ^a^	RT (min)	MS^2^ Mass Spectrum	[M − H]^−^	Molecular Formula	Proposed Compound
1	10.29	Figure 2B	301.2167	C_20_H_30_O_2_	EPA
2	11.26	Figure 2C	277.2168	C_18_H_30_O_2_	ALA
3	12.19	Figure 2D	327.2279	C_22_H_32_O_2_	DHA
4	13.91	Figure 2E	303.2325	C_20_H_32_O_2_	ARA
5	15.66	Figure 2F	279.2287	C_18_H_32_O_2_	LA
6	22.53	Figure 2G	281.2446	C_18_H_34_O_2_	OA

^a^ The number of chromatographic peak in HPLC chromatogram (Figure 1C); RT: the retention time of chromatographic peaks in HPLC; EPA: eicosapentaenoic acid; ALA: α-linolenic acid; DHA: docosahexaenoic acid; ARA: arachidonic acid; LA: linoleic acid; OA: oleic acid.

**Table 3:** The relative correction factors (RCFs) of five unsaturated fatty acids (UFAs) under different injection volumes.

Injection Volume (μL)	F_LA/EPA_	F_LA/ALA_	F_LA/DHA_	F_LA/ARA_	F_LA/OA_
6	2.4906	1.4109	3.2059	2.3822	0.2053
8	2.4991	1.4144	3.2149	2.3760	0.2022
10	2.5257	1.4303	3.2661	2.4051	0.2013
12	2.5410	1.4366	3.2751	2.3968	0.1980
14	2.5631	1.4482	3.3205	2.4180	0.1982
18	2.6097	1.4731	3.3517	2.4335	0.1939
Mean	2.5382	1.4356	3.2724	2.4019	0.1998
RSD (%)	1.73	1.60	1.75	0.90	1.99

EPA: eicosapentaenoic acid; ALA: α-linolenic acid; DHA: docosahexaenoic acid; ARA: arachidonic acid; LA: linoleic acid; OA: oleic acid; RSD: relative standard deviation; F_LA/EPA_, F_LA/ALA_, F_LA/DHA_, F_LA/ARA_, and F_LA/OA_ are the corresponding RCF of EPA, ALA, DHA, ARA, and OA, respectively.

**Table 4:** The robustness test results of relative correction factors (RCF) for five unsaturated fatty acids (UFAs) by Taguchi design–static design.

HPLC Instruments	Chromatographic Columns	F_LA/LA_	F_LA/EPA_	F_LA/ALA_	F_LA/DHA_	F_LA/ARA_	F_LA/OA_
Agilent-UVD	Agilent	1	2.5228	1.4316	3.2654	2.4059	0.1999
Agilent-UVD	Waters	1	2.5132	1.4023	3.2009	2.4014	0.2031
Agilent-UVD	Venusil	1	2.5058	1.4180	3.1377	2.3661	0.2003
Agilent-DAD	Agilent	1	2.5104	1.4052	3.1953	2.3889	0.1994
Agilent-DAD	Waters	1	2.5033	1.4023	3.2901	2.4105	0.1973
Agilent-DAD	Venusil	1	2.5123	1.4193	3.2195	2.4190	0.1983
Waters-E2695	Agilent	1	2.5916	1.4329	3.2130	2.4395	0.1939
Waters-E2695	Waters	1	2.5412	1.4032	3.2014	2.4511	0.2031
Waters-E2695	Venusil	1	2.4994	1.3857	3.1959	2.3498	0.1942
Mean		1	2.5222	1.4112	3.2132	2.4036	0.1988
RSD (%)		0	1.08	1.03	1.28	1.27	1.58

EPA: eicosapentaenoic acid; ALA: α-linolenic acid; DHA: docosahexaenoic acid; ARA: arachidonic acid; LA: linoleic acid; OA: oleic acid; RSD: relative standard deviation; F_LA/EPA_, F_LA/ALA_, F_LA/DHA_, F_LA/ARA_, and F_LA/OA_ are the corresponding RCF of EPA, ALA, DHA, ARA, and OA, respectively; RSD: relative standard deviation.

**Table 5:** The content of six principal unsaturated fatty acids (UFAs) in 15 batches of *Oviductus Ranae* determined based on external standard method (ESM) and quantitative analysis of multi-components by single-marker (QAMS) methods.

Samples	LA (mg/g)	EPA (mg/g)		ALA (mg/g)		DHA (mg/g)		ARA (mg/g)		OA (mg/g)	
	ESM	ESM	QAMS	ESM	QAMS	ESM	QAMS	ESM	QAMS	ESM	QAMS
S1	0.9628	0.1817	0.1808	0.4773	0.4755	0.0889	0.0888	0.4097	0.4090	4.8744	4.9100
S2	0.8547	0.0878	0.0874	0.2758	0.2747	0.0436	0.0435	0.2228	0.2231	3.6959	3.7229
S3	0.9825	0.1147	0.1141	0.1730	0.1724	0.0740	0.0738	0.4689	0.4696	4.6060	4.6397
S4	1.2238	0.2603	0.2590	0.4534	0.4517	0.1522	0.1519	0.6240	0.6248	4.2444	4.2754
S5	0.8017	0.1406	0.1399	0.3770	0.3756	0.0692	0.0690	0.3089	0.3093	3.8682	3.8966
S6	1.2719	0.2103	0.2093	0.4254	0.4239	0.1132	0.1130	0.5726	0.5734	4.8270	4.8623
S7	1.1836	0.2400	0.2388	0.5267	0.5248	0.1327	0.1324	0.5485	0.5492	4.6834	4.7176
S8	1.2175	0.1605	0.1597	0.2909	0.2898	0.1119	0.1117	0.5340	0.5347	3.7866	3.8143
S9	0.7895	0.1427	0.1420	0.1769	0.1762	0.0635	0.0634	0.3990	0.3994	2.9387	2.9602
S10	1.1225	0.1303	0.1296	0.5456	0.5435	0.0747	0.0746	0.3585	0.3590	4.3237	4.3553
S11	0.5216	0.1035	0.1030	0.1328	0.1323	0.0585	0.0584	0.2681	0.2684	2.4207	2.4384
S12	1.2792	0.2292	0.2281	0.2182	0.2174	0.1307	0.1305	0.7264	0.7274	3.3139	3.3381
S13	0.6013	0.1135	0.1129	0.1369	0.1364	0.0547	0.0546	0.3294	0.3299	2.0079	2.0226
S14	0.8133	0.2154	0.2143	0.1958	0.1951	0.0927	0.0925	0.4232	0.4237	2.3952	2.4127
S15	1.0127	0.2919	0.2904	0.3040	0.3029	0.1739	0.1736	0.6477	0.6486	2.4948	2.5131
Si		0.999999987		0.999999992		0.999999930		0.999999780		0.999999999	

EPA: eicosapentaenoic acid; ALA: α-linolenic acid; DHA: docosahexaenoic acid; ARA: arachidonic acid; LA: linoleic acid; OA: oleic acid; Si is the cosine coefficient of the results calculated by the ESM and the QAMS method.

**Table 6:** The detailed information of 15 batches of *Oviductus Ranae* from different collection locations.

NO.	Collection Location	Longitude (° E)	Latitude (° N)	Collection Data
S1	Fusong, Baishan, Jilin	127.29	42.51	2016.12
S2	Huadian, Jilin, Jilin	126.78	43.16	2016.12
S3	Jiangyuan, Baishan, Jilin	126.92	42.08	2016.12
S4	Jingyu, Baishan, Jilin	127.12	42.70	2015.11
S5	Liuhe, Tonghua, Jilin	125.76	42.29	2016.12
S6	Huadian, Jilin, Jilin	126.88	42.86	2016.12
S7	Panshi, Jilin, Jilin	126.21	43.33	2015.11
S8	Huadian, Jilin, Jilin	127.10	42.96	2016.12
S9	Jingyu, Baishan, Jilin	126.52	42.10	2016.12
S10	Helong, Yanbian, Jilin	129.01	42.55	2016.03
S11	Huadian, Jilin, Jilin	126.75	43.33	2016.12
S12	Huadian, Jilin, Jilin	126.68	43.27	2016.12
S13	Jian, Tonghua, Jilin	126.24	41.21	2016.01
S14	Antu, Yanbian, Jilin	128.78	43.20	2016.12
S15	Dunhua, Yanbian, Jilin	129.10	43.43	2016.03

**Table 7:** Two control factors (HPLC instrument and column) and their three level settings.

Levels	Factor-1 (HPLC Instruments)	Factor-2 (Chromatographic Columns)
Level-1	Agilent-UVD	Agilent
Level-2	Agilent-DAD	Waters
Level-3	Waters-E2695	Venusil

## Data Availability

The data presented in this study are available in the article and Supplementary Materials.

## Supplementary Materials

The following are available online. Figure S1: The calibration curve of unsaturated fatty acid (UFA) standards, Figure S2: The extracted ion chromatograms (EICs) of unsaturated fatty acid (UFA) standards, Figure S3: The extracted ion chromatograms (EICs) of *Oviductus Ranae* sample, Table S1: The response results for S/N (Delta 1) and means (Delta 2), Table S2: The results of analysis of variance (ANOVA) for S/N and means, Table S3: The relative error between ESM and QAMS results of 15 batches of *Oviductus Ranae* samples, Table S4: The details of the HPLC chromatograph used in this work, Table S5: The details of the chromatographic columns used in this work.

## Sample Availability

Samples of the compounds are available from the authors.
