# Supplementary material for: Exploitation of HPLC Analytical Method for Simultaneous Determination of Six Principal Unsaturated Fatty Acids in Oviductus Ranae Based on Quantitative Analysis of Multi-Components by Single-Marker (QAMS)
Source: Molecules. 2021 Jan 18;26(2):479. doi: 10.3390/molecules26020479 (PMC7831056; doi:10.3390/molecules26020479)
Supplement: Supplementary file 1 [file molecules-26-00479-s001.pdf]

Exploitation of HPLC Analytical Method for Simultaneous Determination of Six Principal Unsaturated Fatty Acids in *Oviductus Ranae* based on Quantitative Analysis of Multi-components by Single-marker (QAMS)

Shihan Wang [1,*], Yuanshuai Gan [2], Hong Kan [1], Xinxin Mao [1] and Yongsheng Wang [2,*]

[1] College of Chinese Medicine Materials, Jilin Agricultural University, Changchun, Jilin 130118, China; shihanw@jlau.edu.cn (S.W.); kanhong@vip.163.com (H.K.); maomaoheniunai@126.com (X.M.)

[2] School of Pharmaceutical Sciences, Jilin University, Changchun, Jilin 130021, China; ganys18@mails.jlu.edu.cn (Y.G.); wys@jlu.edu.cn (Y.W.)

* Correspondence: shihanw@jlau.edu.cn (S.W.); wys@jlu.edu.cn (Y.W.)

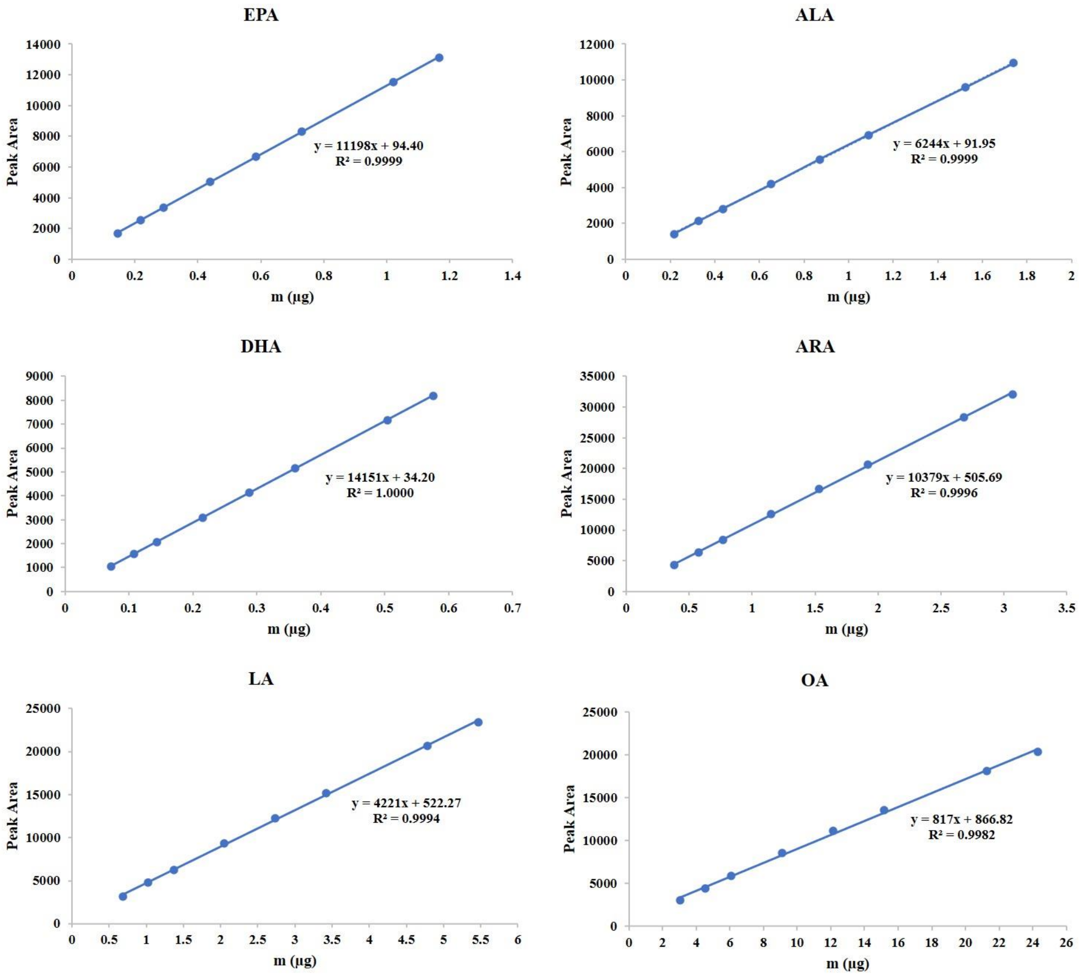

Figure S1. The calibration curve of unsaturated fatty acid (UFA) standards.

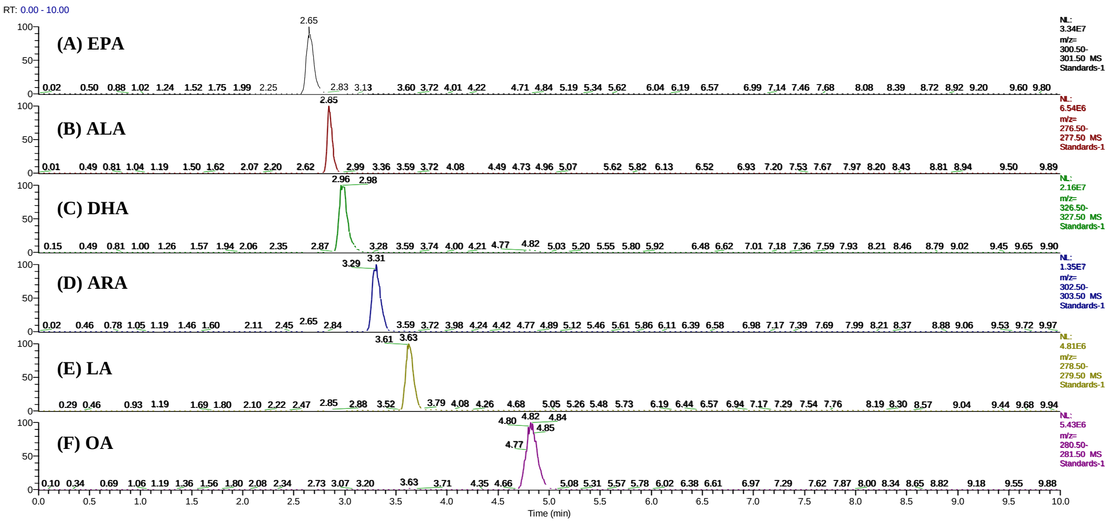

Figure S2. The extracted ion chromatograms (EICs) of unsaturated fatty acid (UFA) standards.

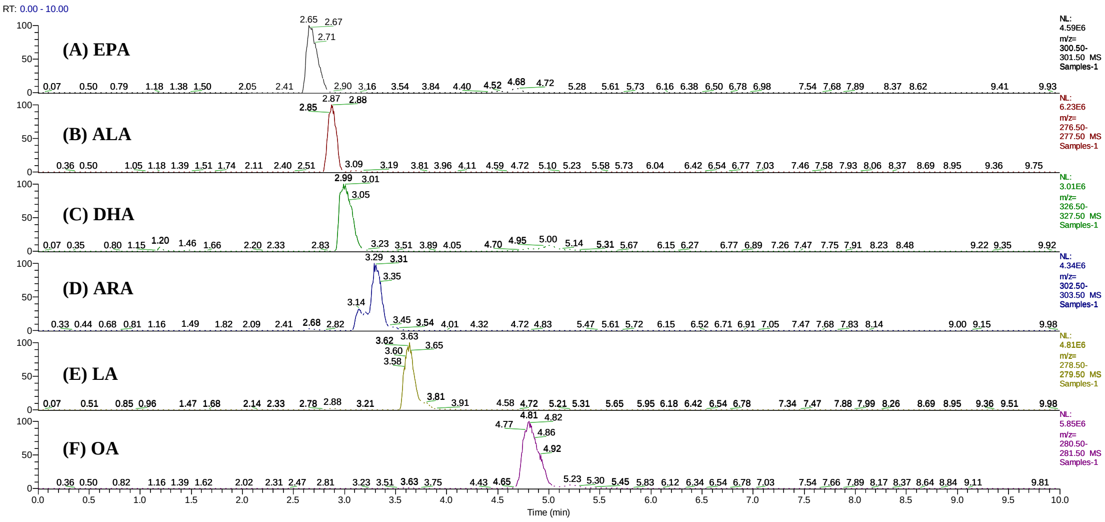

Figure S3. The extracted ion chromatograms (EICs) of *Oviductus Ranae* sample.

Table S1. The response results for S/N (Delta 1) and means (Delta 2)

Source	HPLC Instruments	Chromatographic Columns
S/N (Delta 1)	0.059	0.056
Rank	1	2
Mean (Delta 2)	0.007	0.021
Rank	2	1

Table S2. The results of analysis of variance (ANOVA) for S/N and means

Source	DF	p-value (S/N)	p-value (Mean)
HPLC Instruments	2	0.535	0.843
Chromatographic Columns	2	0.565	0.291

Table S3. The relative error between ESM and QAMS results of 15 batches of *Oviductus Ranae* samples

Samples	LA	EPA (%)	ALA (%)	DHA (%)	ARA (%)	OA (%)
S1	N/A	-0.50	-0.38	-0.11	0.17	0.73
S2	N/A	-0.46	-0.40	-0.23	0.13	0.73
S3	N/A	-0.52	-0.35	-0.27	0.15	0.73
S4	N/A	-0.50	-0.37	-0.20	0.13	0.73
S5	N/A	-0.50	-0.37	-0.29	0.13	0.73
S6	N/A	-0.48	-0.35	-0.18	0.14	0.73
S7	N/A	-0.50	-0.36	-0.23	0.13	0.73
S8	N/A	-0.50	-0.38	-0.18	0.13	0.73
S9	N/A	-0.49	-0.40	-0.16	0.10	0.73
S10	N/A	-0.54	-0.38	-0.13	0.14	0.73
S11	N/A	-0.48	-0.38	-0.17	0.11	0.73
S12	N/A	-0.48	-0.37	-0.15	0.14	0.73
S13	N/A	-0.53	-0.37	-0.18	0.15	0.73
S14	N/A	-0.51	-0.36	-0.22	0.12	0.73
S15	N/A	-0.51	-0.36	-0.17	0.14	0.73

Table S4. The details of the HPLC chromatograph used in this work

Models	Detector	Workstation
Agilent-UVD	Ultraviolet detector	Agilent ChemStation
Agilent-DAD	Diode array detector (DAD)	Agilent ChemStation
Waters-E2695	Ultraviolet detector	Empower 3

Table S5. The details of the chromatographic columns used in this work

**Brand**	**Type**	**Length (mm)**	**Aperture (Å)**	**Carbon Load (%)**	**Surface Area (m^2/g)**	**End Capping**
Agilent	TC-C18	250×4.6	130	12	300	Yes
Waters	XBridge-C18	250×4.6	135	18	185	Yes
Venusil	MP-C18	250×4.6	100	18	380	Yes
